# Cyclooxygenase-2 activates EGFR–ERK1/2 pathway via PGE2-mediated ADAM-17 signaling in testosterone-induced benign prostatic hyperplasia

**DOI:** 10.1007/s10787-022-01123-7

**Published:** 2022-12-31

**Authors:** Mohammed E. Abo-El Fetoh, Maha M. Abdel-Fattah, Wafaa R. Mohamed, Laila A. A. Ramadan, Hassan Afify

**Affiliations:** 1 Department of Pharmacology and Toxicology, Faculty of Pharmacy, Egyptian Russian University, Cairo, Egypt; 2 Department of Pharmacology and Toxicology, Faculty of Pharmacy, Beni-Suef University, Beni-Suef, 62514 Egypt

**Keywords:** Benign prostatic hyperplasia, COX-2, PGE2, ADAM-17 (TACE), EGFR–ERK1/2, Celecoxib

## Abstract

**Objective and design:**

Prostatic inflammation is the driving force in benign prostatic hyperplasia (BPH). This work investigated the potential modulatory effect of COX-2 inhibition on ADAM-17/EGFR/ERK1/2 axis.

**Materials or subjects:**

Adult male Wistar rats were used.

**Treatment:**

Celecoxib (10 and 20 mg/kg; i.p.) was injected i.p. daily for three weeks. Testosterone (TST) (3 mg/kg; s.c.) was used to induce BPH.

**Methods:**

Prostatic inflammation and hyperplasia were assessed by organ weight and histopathology. Inflammatory mediators were measured using ELISA technique. Protein analysis was performed using western blotting and immunohistochemistry. Gene expression analysis was performed using qRT-PCR. Statistical analyses included one-way ANOVA and Tukey’s multiple comparison test.

**Results:**

Testosterone-treated rats had a marked increase in COX-2, prostate weight, and index. Moreover, TST-induced COX-2 was inferred from cytoskeletal changes and was attributable to the overexpression of PGE2, NF-κB (p65), and IL-6. COX-2-derived PGE2 increased the activity of ADAM-17, TGF-α, and TNF-α. Consequently, EGFR–ERK1/2 pathway was over-activated, disrupting anti-apoptotic Bcl-2, cyclin D1, and pro-apoptotic Bax. Celecoxib reversed these effects.

**Conclusion:**

COX-2 stimulates the ERK1/2 pathway via PGE2–ADAM-17-catalyzed shedding of TGF-α in testosterone-induced BPH. The results indicate a functional correlation between inflammation and hyperplasia in BPH.

## Introduction

Cyclooxygenase-2 (COX-2) is of major interest in several inflammatory disorders. COX-2 is an inducible cyclooxygenase isozyme that responds inappropriately to inflammatory stimuli. Unlike COX-2, COX-1 is normally expressed throughout the tissues and is required for optimal homeostasis. Generally, several factors may be implicated in the activation of COX-2, for example, nuclear factor kappa-light-chain enhancer of activated B cells (NF-κB), tumor necrosis factor alpha (TNF-α), transforming growth factor alpha (TGF-α), and epidermal growth factor (EGF) (Morita 2002).

Benign prostatic hyperplasia (BPH) is a prevalent, aging-related chronic condition. BPH is seen as a clear-cut example of both inflammation and hyperplasia. It is characterized by the production of chemokines and cytokines, which promote inflammation. Prostaglandin E2 (PGE2) levels increase along with COX-2 levels, ultimately resulting in prostate cell proliferation (Chughtai et al. 2011). Despite the fact that COX-2 is highly expressed in BPH, it is not known how COX-2 causes prostatic enlargement (Robert et al. 2009). Testosterone (TST) is considered one of the most important risk factors for BPH. Besides being necessary to preserve sexual activity, the accumulated evidence indicates the inverse correlation of TST with age-related disorders in males, such as obesity, diabetes, metabolic syndrome, cardiovascular disorders, and cognitive impairment (Stanworth and Jones 2008). However, there are still some controversies surrounding the initiation and progression of BPH with high or low TST (Saad et al. 2011). Experimental and clinical studies have shown that the administration of anabolic steroid supplements stimulates the enlargement of the prostate in rats (Vargas et al. 2013), guinea pigs (Acosta et al. 2004), and even in bodybuilders (Kanayama et al. 2008, 2010).

Indeed, there is a well-identified interaction between COX-2 and epidermal growth factor receptor (EGFR). On the basis of efficacy, COX-2 inhibitors and EGFR inhibitors have reasonable therapeutic efficacy in non-malignant and malignant disorders (Dannenberg et al. 2005). Increased COX-2 transcription leads to increased synthesis of prostaglandins, especially PGE2, which can trans-activate EGFR signaling. PGE2 has been shown in several studies to increase cell proliferation through trans-activating EGFR signaling (Pai et al. 2002; Buchanan et al. 2003; Shao et al. 2003). Similar to other plasma membrane receptors, EGFRs commonly have an intracellular tyrosine kinase domain, a transmembrane segment, and an extracellular ligand-binding domain (Chen et al. 2016a). In the 1960s, the pivotal role of EGFR was well established in normal development and several disorders (Cohen 1962). Among all EGFR ligands, TGF-α and EGF have the greatest affinity. TGF-α binds to EGFR, resulting in the formation of homo-dimeric complexes, which are quickly followed by phosphorylation of the receptor. Typically, seven signaling molecules transmit the downstream effects of EGFR activation. The most significant pathway triggered by EGFR is ERK–MAPK; this pathway is known to be critical in cellular growth, differentiation, migration, and proliferation (Wee and Wang 2017).

Celecoxib (CXB) is a selective COX-2 inhibitor that has been demonstrated to help patients with BPH who experience nocturia (Falahatkar et al. 2008; Goodarzi et al. 2011). In addition, CXB is approved to be safe for several age-related inflammatory disorders (Goldenberg 1999; Dougados et al. 2001). In the current study, two daily doses of CXB were been selected (i.e., 10 mg/Kg and 20 mg/Kg) that were equivalent to those safe and well-tolerated in humans (Goodarzi et al. 2011; Nair et al. 2018). It is important to remember that CXB is preferred over other COX inhibitors owing to its selectivity for COX-2 and reduced side effects, particularly in elderly patients (Bushman and Jerde 2016). The present study employed CXB prophylactically to guarantee pre-inhibition of COX-2, which specifically impacts the relationship between COX-2 and the EGFR–ERK1/2 pathway in both the initiation and progression of testosterone-induced BPH in rats. Consequently, the main objective of the current work is to investigate the potential modulatory effect of COX-2 inhibition (via CXB) on ADAM-17/EGFR/ERK1/2 axis. Furthermore, this study re-evaluated CXB as a prophylactic approach for BPH and not only a symptomatic treatment for lower urinary tract symptoms induced by BPH.

## Materials and methods

### Materials

**Celecoxib** (**CXB**) was obtained from Pfizer Pharmaceuticals^®^, Cairo, Egypt, freshly dissolved in isotonic saline (Sun et al. 2013). **Testosterone** (**TST**) was obtained from Chemical Industries Development Co.^®^ (CID) Giza, Egypt. It was reconstituted in olive oil to the required concentration (Abo-Youssef et al. 2020).

### Animals

Eight-week-old male Wistar rats (150–200 g body weight) were kept in a controlled environment (22 °C ± 2 °C and constant humidity) under a 12 h light/dark cycle. A standard diet and water were freely available.

### Ethics approval statement

The Institutional Animal Care and Use Committee of Beni-Suef University (**BSU-IACUC-021–198**) authorized the experiments prior to the study. Furthermore, the study protocols followed the National Institutes of Health’s recommendations for the care and use of laboratory animals (Garber 2011). The current animal study adhered to the ARRIVE guidelines (Percie du Sert et al. 2020).

### Experimental design

Rats were randomly allocated to one of five groups; each contained six animals (Festing 2006). All animals were treated for three weeks. The treatment and groups were: the ***control*** group, administered **isotonic saline** (1 mL/kg/day; i.p.) plus 1 mL/kg/day **olive oil** subcutaneously; the ***CXB-20*** group, was received **celecoxib** (**CXB**) (20 mg/kg/day; i.p.) (Sun et al. 2013; Nair et al. 2018) for 3 weeks plus 1 mL/kg/day **olive oil** subcutaneously; ***TST-induced BPH*** group, administered **isotonic saline** (1 mL/kg/day; i.p.) plus **testosterone** (**TST**) (3 mg/kg/day; s.c) for 2 weeks (Maggi et al. 1989; Pandita et al. 1998; Abo-Youssef et al. 2020); the ***CXB-10 plus TST*** group was received **CXB** (10 mg/kg/day; i.p.) (Sun et al. 2013; Nair et al. 2018) for 3 weeks, starting 1 week before **TST** administration (3 mg/kg/day; s.c.); the ***CXB–20 plus TST*** group was administered **CXB** (20 mg/kg/day; i.p.) (Sun et al. 2013; Nair et al. 2018) for three weeks, starting one week before **TST** administration (3 mg/kg/day; s.c.).

At the end of the experiment, the rats were weighed and then anesthetized by **thiopental sodium** (30 mg/kg; i.p) (Egyptian International Pharmaceutical Industries Company [EIPICO]^®^, Tenth of Ramadan City, Egypt.) (Fatehi-Hassanabad et al. 2005). Rats were then sacrificed by decapitated and prostate tissues were quickly dissected out, washed, then weighed. The prostate ventral lobes were divided into four sections. The first section was fixed in neutral buffered formalin (10%) for histological and immunohistochemical examinations. The second section was preserved in lysis buffer containing a protease inhibitor cocktail for western blotting analysis and stored at − 80 °C. The third section was stored in RNA later for use in quantitative real-time polymerase chain reaction analysis (qRT-PCR). The final section was kept at − 80 °C for use in further analysis.

### Assessment of inflammation and hyperplasia in prostatic architecture

From each, the prostate was dissected and immediately weighed. The prostate index (PI) was determined by dividing the prostate weight (PW) by the overall body weight (Abo-Youssef et al. 2020).

Paraffin slices (4 µm) were produced from the fixed prostate samples (10% buffered formal saline) and then stained with hematoxylin and eosin. The tissue slides were then examined under a Leica microscope (Leica Microsystems GmbH^®^, Wetzlar, Germany). All light microscopic examinations and morphometric data utilized the Leica Application module for histological analysis, which was connected to a full HD microscopic imaging system (Leica Microsystems GmbH^®^, Germany) (Culling 1974). Each sample’s mean lining epithelial cell height was determined by scanning six non-overlapping fields from each ventral lobe tissue segment (Said et al. 2015).

### Measurement of inflammatory mediators and metalloproteinase (ADAM-17 or TACE) activity in prostatic tissue using ELISA

In accordance with the manufacturer’s protocol, the assessment of COX-2 (Cusabio^®^, Houston, USA), NF-κB (Elabscience^®^, Houston, USA), PGE2 (Elabscience^®^, Houston, USA), IL-6 (Immuno-Biological Laboratories^®^, Minneapolis, USA), TNF-α (Cloud-Clone Corp.^®^, Texas, USA), ADAM-17 or TACE (Elabscience^®^, Houston, USA), and TGF-α (LifeSpan Biosciences^®^, Houston, USA) in prostatic tissue homogenates was performed using the corresponding rat ELISA kits.

### Western blotting analysis of protein expression of p-EGFR and p-ERK1/2 in prostatic tissue

To determine the total protein content, the Bradford protein assay kit (Bio-Rad^®^, California, USA) was applied to each sample of the homogenized tissues (Bradford 1976). The protein levels of p-ERK1/2, T-ERK1/2, p-EGFR, and T-EGFR were assessed using western blotting. First, SDS lysis buffer was freshly prepared by adding the following components; 10 mM Tris, 100 mM NaCl, 25 mM ethylene-diamine tetra-acetic acid (EDTA), 25 mM ethylene glycol bis(2-aminoethyl) tetra-acetic acid (EGTA), 0.1% sodium dodecyl sulfate (SDS), 2% (v/v) Triton X-100 (pH 7.4), with 1:1000 protease inhibitor cocktail and phosphatase inhibitors (Elnagar et al. 2018; Abo-El Fetoh et al. 2020). Prostatic homogenates were then prepared in SDS buffer and subjected to SDS–polyacrylamide gel electrophoresis and western blotting analysis as described previously (Burnette 1981). To denature the proteins, 50 µg of total protein from each sample was mixed with an equivalent amount of 2 × electrophoresis sample buffer and heated at 95 °C for 10 min. After gel electrophoresis, the proteins were transferred onto polyvinylidene fluoride (PVDF) membranes (Bio-Rad^®^, California, USA) by semi-dry electro-blotting.

#### Primary incubation

Nonspecific binding to the PVDF membranes was initially blocked by incubation of the membrane in 5% non-fat dry milk in Tris-buffered saline containing 0.05% Tween (TBST) for 1 h. Then, the membrane was incubated with the primary antibodies against p-ERK1/2 (1:1000) (Cat# 8544–RRID: AB_11127856), T-ERK1/2 (1:2000) (Cat# 9102–RRID: AB_33074), p-EGFR (1:1000) (Cat# 2234–RRID: AB_331701), and T-EGFR (1:1000) (Cat# 2646–RRID:AB_2230881) (all from Cell Signaling Technology^®^, Massachusetts, USA) diluted in 1 × TBST buffer overnight at 4 °C. Four washes (each for 10 min) with 1 × TBST buffer were performed.

#### Secondary incubation

The PVDF membrane was then incubated with secondary antibody coupled to horseradish peroxidase (Cell Signaling Technology^®^, Massachusetts, USA, Cat# 7074–RRID: AB_2099233) diluted in 1 × TBST buffer (1:3000) for 30 min.

#### Detection

Enhanced chemiluminescence (ECL) solution (Perkin Elmer, Waltham^®^, Massachusetts, USA) was used to detect the signals with ChemiDoc imager (Bio-Rad^®^, California, USA) and the band intensity was measured using ImageLab^®^ analysis software (Version 6.1.0 build 7, California, USA). T-ERK1/2 and T-EGFR were considered as loading control proteins, as discussed previously (Taylor et al. 2013; Abo-El Fetoh et al. 2020).

### Detection of Cyclin D1 by immunohistochemistry

Prostatic tissue sections were dried, deparaffinized, and rehydrated before boiling in citrate buffer (pH 6.0) for 10 min. The tissue sections were gently washed for 2 h in TBS containing 5% bovine serum albumin (BSA). Anti-Cyclin D1 antibody (1:250) (Cell Signaling Technology^®^, Massachusetts, USA, Cat# 55506–RRID: AB_2827374) was incubated with the tissue sections overnight at 4 °C. After careful washing of the slides in TBS with 5% BSA, the slides were incubated with secondary antibody (Cell Signaling Technology^®^, Massachusetts, USA, Cat# 8114–RRID: AB_10544930), washed, and then treated with diaminobenzidine (DAB) for 15 min and washed in PBS. The slides were stained with hematoxylin, cleared in xylene, and dehydrated for microscopic examination (Kalyuzhny 2016). ImageLab^®^ analysis software (Version 6.1.0 build 7, California, USA) was used to acquire the images and quantify the images. Six non-overlapping fields were randomly selected and scanned from each ventral lobe tissue section from each sample of immune-stained tissue sections for the determination of the mean percentage of immunohistochemical expression levels of cyclin D1 (Hsu et al. 2021).

### Analysis of bax and Bcl-2 by real-time polymerase chain reaction (qRT-PCR)

qRT-PCR was used to measure the mRNA expression of pro-apoptotic Bax and anti-apoptotic Bcl-2. RExPrimer^®^ was used to design primers (Piriyapongsa et al. 2009). The nucleotide sequences of the primers are shown in **Table** 1. Total RNA was extracted from prostatic tissues using TRIzol reagent Mini kit (Invitrogen^®^, Carlsbad, CA) in accordance with the manufacturer’s instructions. The mRNA expression of pro-apoptotic Bax and anti-apoptotic Bcl-2 was detected using a mixture of SYBR Green mastermix and Hot Start Taq RNA Polymerase (Jena Bioscience^®^, Jena, Germany). Based on the manufacturer’s guidelines, the following thermo-cycling conditions were applied in standard mode: 10 min at 95ºC, followed by 40 cycles of amplification (95ºC for 15 s, 57ºC for 45 s, and 72ºC for 45 s). ABI Prism^®^ 7000 SDS Software was used to analyze the data. Finally, expression of each gene (i.e., number of copies) was calculated from the standard curve provided by each kit. The median of the RNA expression was calculated and used as a threshold to differentiate between the higher and lower expression within the factor groups. The mRNA expression was normalized to the expression of the housekeeping gene β-actin (Chen et al. 2016b).

**Table 1:** Primers' sequence for pro-apoptotic Bax, anti-apoptotic Bcl-2, and β-actin

Gene	Primer sequence	
	Forward	Reverse
Bax	5–CGG CGA ATT GGA GAT GAA CTGG–3	5–CTA GCA AAG TAG AAG AGG GCA ACC–3
Bcl-2	5–GTG GAT GAC TGA GTA CCT–3	5–CCA GGA GAA ATC AAA CAGAG–3
β-Actin	5–AAG ATC CTG ACC GAG CGTGG–3	5–CAG CAC TGT GTT GGC ATA GAGG–3

### Data and statistical analysis

Statistical analysis was conducted only for a group size of ≥ 5. Statistical significance was calculated using one-way ANOVA. Additionally, differences between groups were analyzed using Tukey’s multiple comparisons test. A probability (P) value of < 0.05 was considered to indicate statistical significance. All values are presented as the mean ± standard error of the mean (SEM). Data management and analysis were performed using GraphPad Prism^®^ software for Windows (Version 9.2.0.332–San Diego, USA).

## Results

### The main hallmark of inflammation in testosterone (TST)-induced BPH is COX-2

**Fig. 1:**
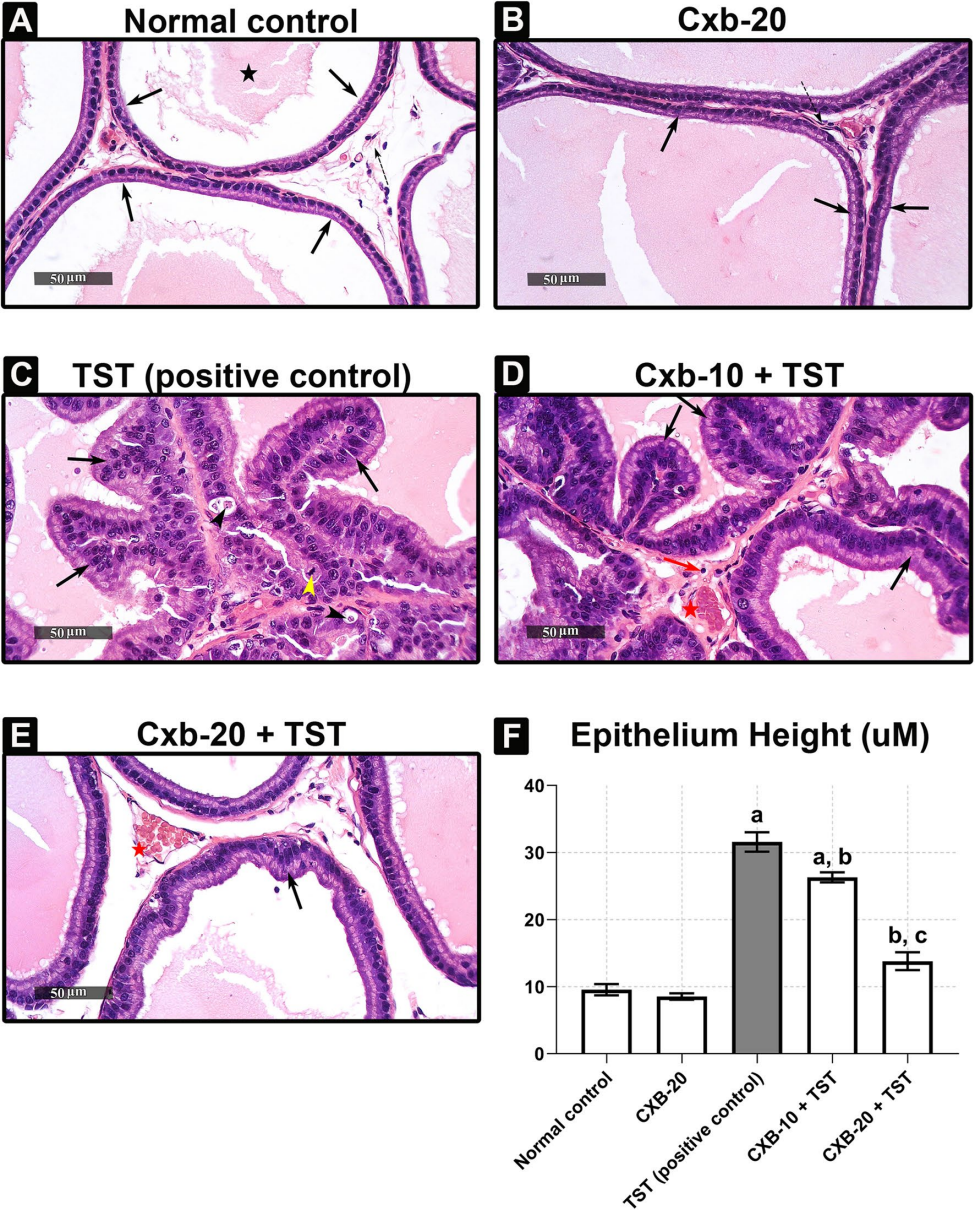
**TST-induced COX-2 clearly deteriorates the histological architecture of prostate in BPH. Histological examination of rat’s prostate:** **a** Sections of **control rats** show normal histo-architecture of the ventral prostates. **b** **Treatment with 20 mg of CXB alone** showing unremarkable changes to control group. **c** **Induction of BPH with TST** exhibiting hypertrophy with increased epithelial thickness and intra-luminar projections and inflammatory infiltrate composed mainly of lymphocytes. **d** **Treatment with 10 mg of CXB** showing almost the same records as **TST-induced group** allover most of glandular acini with few scattered apparent intact acini in between. **e** **Testosterone co-treated with 20 mg/kg CXB** showing marked reduction in hypertrophy and hyperplasia. **f** **Thickness of the basal epithelium cells** showing significant reduction of cell membrane thickness with **CXB-20–treated** compared to **TST-treated rats**. The data are provided as mean ± SEM (*n* = 6). ^a^significant versus *Control*; ^b^significant versus *TST*; ^c^significant versus *CXB-10* + *TST* at *P* < 0.05. *CXB* Celecoxib; *TST* Testosterone. (Scale bar = 50 μm)

**Fig. 2:**
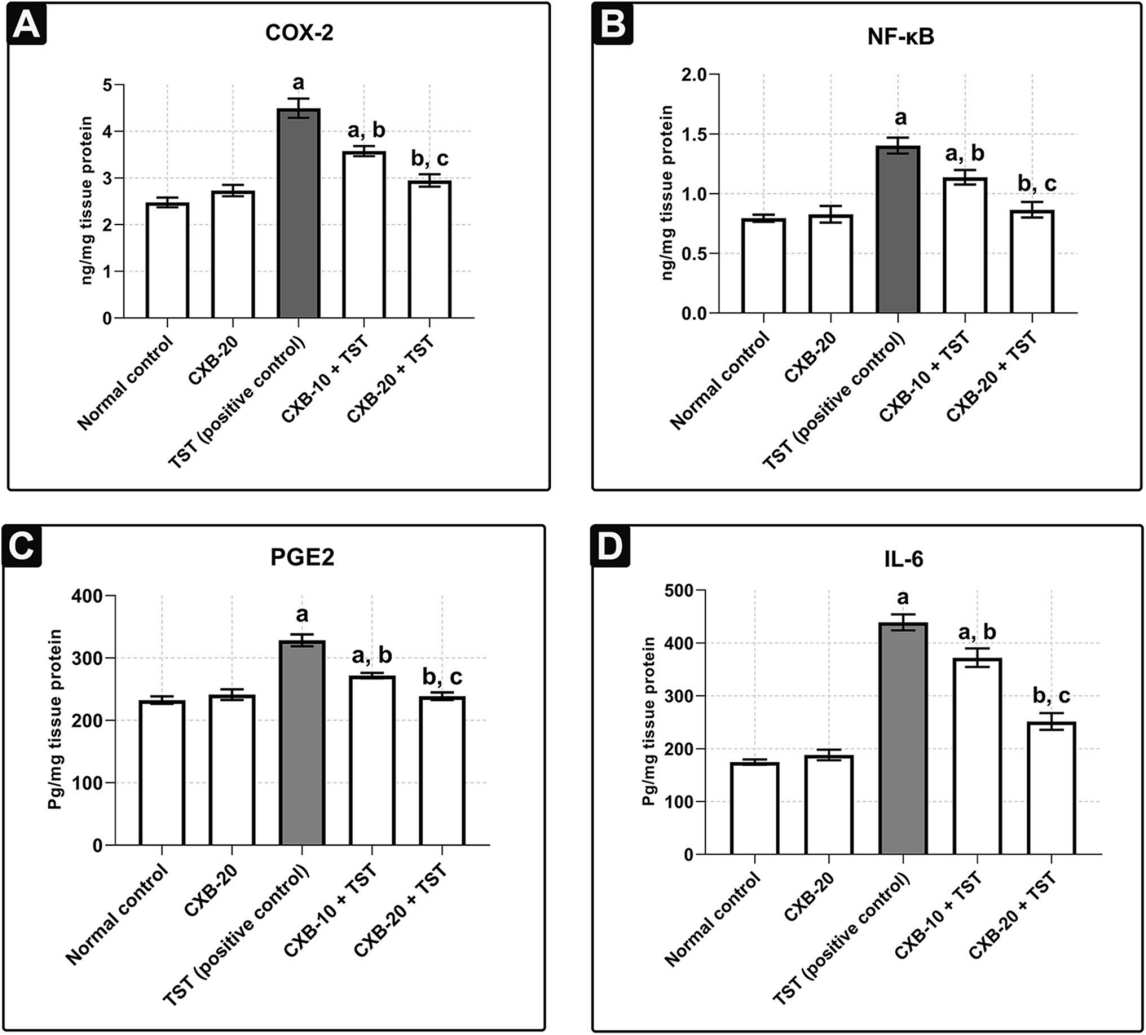
**Summary of inflammatory markers induced by TST in BPH and the subsequent effect of celecoxib (CXB) on these markers: Evaluation of inflammatory markers using quantitative ELISA technique:** Prostate content of **COX-2** (ng/mg) **a**, **NF-κB** (p65) (ng/mg) **b**, **PGE2** (Pg/mg) **c** and **IL-6** (Pg/mg) **d** are significantly improved with co-administration of CXB. All inflammatory markers are further normalized with higher dose. The data are provided as mean ± SEM (*n* = 6). ^a^significant versus *Control*; ^b^significant versus *TST*; ^c^significant versus *CXB-10* + *TST* at *P* < 0.05. *CXB* Celecoxib, *TST* Testosterone; *NF-κB* (p65) Nuclear factor kappa-light-chain-enhancer of activated B cells, *IL-6* Interleukin 6, *COX-2* Cyclooxygenase enzyme isoform 2, *PGE2* Prostaglandin E2

First, we confirmed the occurrence of BPH as a result of the subcutaneous injection of testosterone (TST). As shown in **Table** 2, the net PW was clearly increased in the **TST-treated group** (by **104%**) above the **control**. Interestingly, PI was significantly raised in the **TST-treated group** (by **93%**) above the **control**. In addition, we monitored the effect of TST-induced COX-2 on the cellular architecture of the prostate gland. The cross-sections and scoring analysis of **TST-treated rats** (**Fig.** 1**c** and **Table** 3) indicated massive congestion of the microvasculature (**scoring +  +  + +**) within prostate tissue. Furthermore, the presence of edema is apparent (**scoring + +**), with inflammatory cells in the perivascular compartment (**scoring + +**) and marked proliferation of the acinar lining epithelium (**scoring +  +  + +**). The abnormal histopathological changes were restored in the absence of COX-2, as shown in **Fig.** 1**d** and **Fig.** 1**e**. In addition, the quantitative analysis (**Fig.** 1**f**) revealed that the epithelium height was significantly increased by **three-fold** above the **control**. Other evidence presented in **Fig.** 2**a** shows that TST induced the highest level of COX-2 expression (by **81%**) above the **control**. Compared with **TST-treated rats**, the pre-administration of 20 mg/kg/day celecoxib (**CXB-20 plus TST**) clearly attenuated COX-2 activity by **34%**, whereas 10 mg/kg/day celecoxib (**CXB-10 plus TST**) showed only a small improvement.

**Table 2:** TST-induced COX-2 enhances prostate growth in BPH

	Prostate weight (mg)	Prostate index
Normal control	465.0 ± 36.63	1.873 ± 0.15210
CXB-20	467.5 ± 46.61	1.820 ± 0.15340
TST (positive control)	947.5 ± 78.89 ^a^	3.617 ± 0.3259 ^a^
CXB-10 + TST	677.5 ± 50.89 ^b^	2.815 ± 0.06248 ^a, b^
CXB-20 + TST	595.0 ± 35.24 ^b^	2.010 ± 0.05774 ^b, c^

The data are provided as mean ± SEM, (*n* = 6)

^a^ significant versus ***Control***; ^b^ significant versus ***TST***; ^c^ significant versus ***CXB-10 + TST*** at *P* < 0.05

*CXB* Celecoxib, *TST* Testosterone

**Table 3:** COX-2 inhibition ameliorates both prostatic inflammation and hyperplasia in BPH

	Congestion	Inflammation	Edema	Mitosis	Apoptosis	Hyperplasia
Normal control	** + **	**−**	**−**	**−**	**−**	**−**
CXB-20	**−**	**−**	**−**	**−**	**−**	**−**
TST (positive control)	** + + + + **	** + + **	** + + **	** + + + **	** + + + **	** + + + + **
CXB-10 + TST	** + + + **	** + + **	** + + **	** + + **	** + + **	** + + + **
CXB-20 + TST	** + + **	**−**	**−**	**−**	**−**	**−**

The data are provided as mean ± SEM (*n* = 6)

**(−)** minimal, **( +)** low, **(+ +)** mild, **(+ + +)** moderate, and **(+ +  + +)** severe

*CXB* Celecoxib, *TST* Testosterone

A positive correlation was found between COX-2 and PW, which was apparent the when COX-2 inhibitor (10 and 20 mg/kg/day CXB) was employed. As shown in **Table** 2, the concurrent administration of 20 mg/kg/day celecoxib (**CXB-20 plus TST**) efficiently decreased PW (by **37%**) and PI (by **44%**) compared with **TST-treated rats**. However, 10 mg/kg/day celecoxib (**CXB-10 plus TST**) caused a smaller improvement. In addition, the scoring values (**Table** 3) were alleviated by the COX-2 inhibitor. Interestingly, the inhibition of COX-2 (by 10 and 20 mg/kg/day CXB) exerted a significant reduction in the thickness of the cellular epithelium compared with **TST-treated rats**.

### Testosterone (TST) activates the NF-κB inflammatory pathway in BPH

Next, we evaluated the involvement of the NF-κB inflammatory pathway in BPH. As shown in **Fig.** 2**b**, there was a marked difference in the activity of NF-κB in **TST-treated rats** compared with the **control rats**. Levels of NF-κB were significantly elevated in **TST-treated rats** (by **76%**) compared with **control rats**. In addition, it has been well established that celecoxib (CXB) causes strong inhibition of NF-κB (Funakoshi-Tago et al. 2008). Compared with **TST-treated rats**, 20 mg/kg/day celecoxib (**CXB-20 plus TST**) reduced levels of NF-κB by **38%**, whereas 10 mg/kg/day celecoxib (**CXB-10 plus TST**) resulted in only a minor improvement, indicating the dose-dependent effects of CXB.

### COX-2-derived PGE2 is involved in testosterone (TST)-induced BPH

As illustrated in **Fig.** 2**c**, **TST-induced rats** had a significant increase in PGE2 activity (by **41%**) compared with **control rats**. Compared with **TST-induced rats**, the absence of COX-2 in the 20 mg/kg/day celecoxib (**CXB-20 plus TST**) group clearly abolished PGE2 activity (by **27%**), whereas 10 mg/kg/day celecoxib (**CXB-10 plus TST**) had a smaller effect.

### Testosterone (TST)-induced COX-2 upregulates interleukin 6 in BPH

Interleukin 6 (IL-6) levels were clearly increased in **TST-induced rats** (by **1.5 times**) compared with **control rats** (**Fig.** 2**d**). Compared with **TST-treated rats**, the concomitant administration of 20 mg/kg/day celecoxib (**CXB-20 plus TST**) strongly suppressed IL-6 levels (by **43%**), whereas 10 mg/kg/day celecoxib (**CXB-10 plus TST**) caused a minor suppression.

### Testosterone (TST)-induced COX-2 is critical for ERK1/2 phosphorylation in BPH

**Fig. 3:**
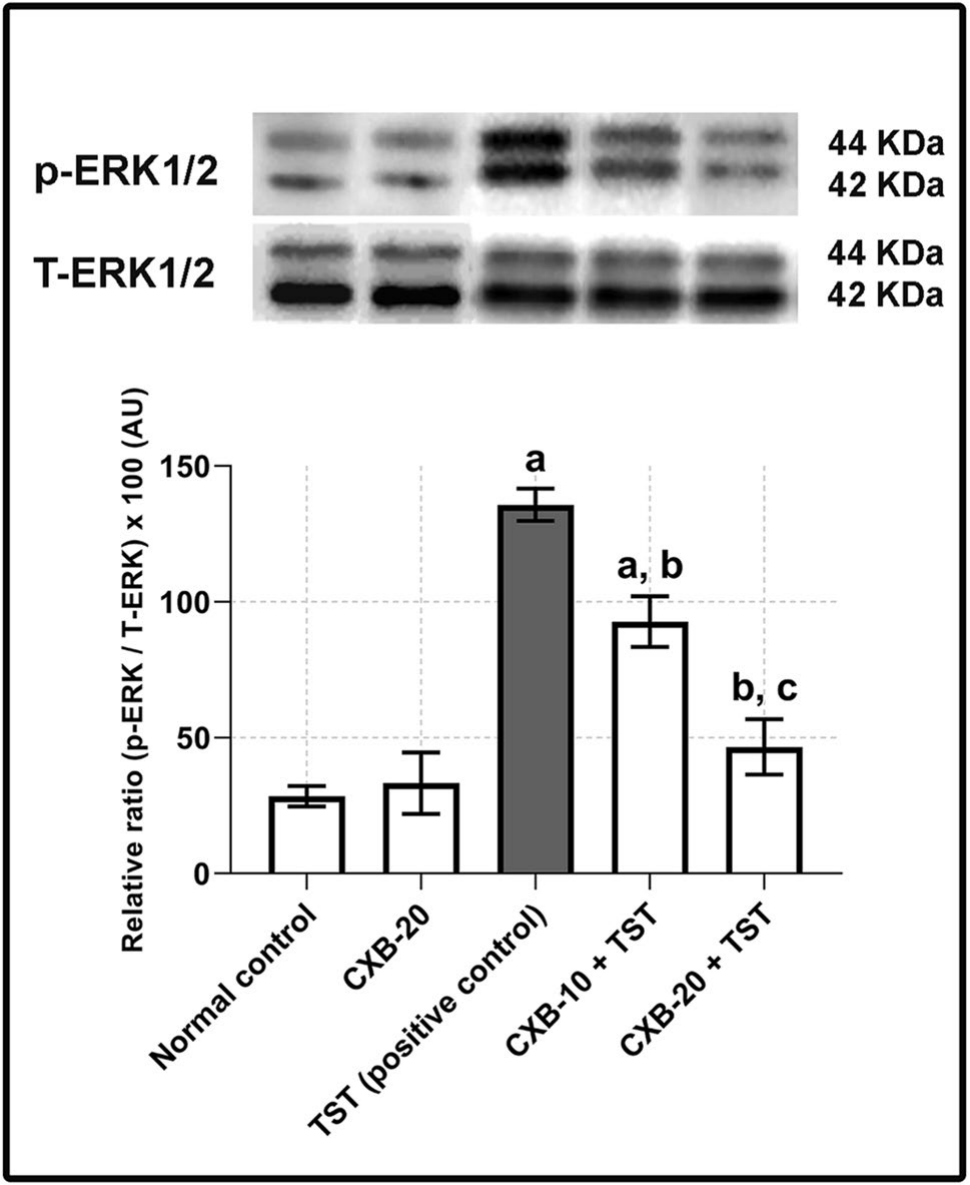
**TST-induced COX-2 is critical for phosphorylation of ERK1/2 in BPH. Assessment of phosphorylation of ERK1/2 using western blotting technique:** Prostate content of p-ERK1/2/T-ERK1/2 is significantly improved with co-administration of CXB. It is apparent that inhibition of COX-2 is critical for decreasing phosphorylation of ERK1/2. The data are provided as mean ± SEM (*n* = 6). ^a^ significant versus *Control*; ^b^ significant versus *TST*; ^c^ significant versus *CXB-10* + *TST* at *P* < 0.05. *CXB* Celecoxib, *TST* Testosterone, *ERK1/2* Extracellular signal-regulated kinase 1 and 2

In this experiment, the mechanistic relevance between COX-2 and prostatic hyperplasia was considered. Several reports have considered the crosstalk between COX-2 and the EGFR–ERK1/2 pathway (Pai et al. 2002; Shao et al. 2003). Therefore, the potential effect of TST-induced COX-2 on the ERK1/2 pathway was examined. In line with high COX-2 expression (**Fig.** 2**a**), the results of the western blotting analysis in **Fig.** 3 revealed that **TST-induced rats** had a marked elevation in ERK1/2 phosphorylation (**four times** higher than the **control rats**).

Furthermore, the significant inhibitory effects of COX-2 inhibitors support COX-2-mediated ERK1/2 activation (in the **CXB-10 plus TST** and **CXB-20 plus TST** groups). The quantitative analysis presented in **Fig.** 3 reveals an obvious reduction in ERK1/2 phosphorylation (by **66%**) in 20 mg/kg/day celecoxib (**CXB-20 plus TST**), with only a minor effect in the 10 mg/kg/day celecoxib (**CXB-10 plus TST**) compared with TST-induced rats.

### The metalloproteinase (ADAM-17 or TACE) is involved in COX-2-induced ERK1/2 phosphorylation in testosterone (TST)-treated animals

**Fig. 4:**
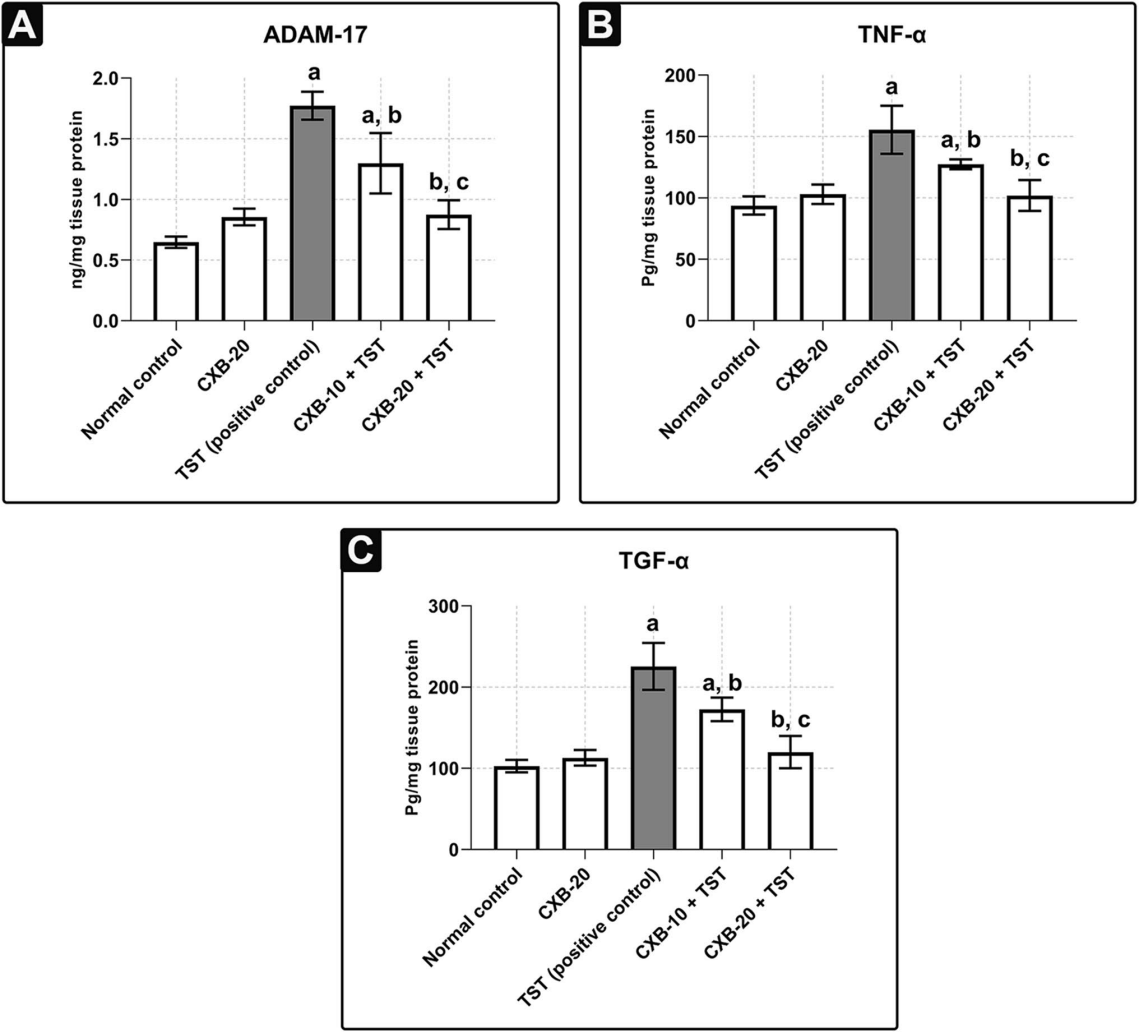
**TST-induced COX-2 triggers metalloproteinase activity of ADAM-17 (TACE) and its substrates in BPH. Evaluation of metalloproteinase activity of ADAM-17 and its substrates using quantitative ELISA technique:** Prostate content of **ADAM-17** or **TACE** (ng/mg) **a**, **TNF-α** (ng/mg) **b** and **TGF-α** (Pg/mg) **c** are significantly improved with co-administration of CXB. The data are provided as mean ± SEM (*n* = 6). ^a^significant versus *Control*; ^b^significant versus *TST*; ^c^significant versus *CXB-10* + *TST* at *P* < 0.05. *CXB* Celecoxib, *TST* Testosterone; *ADAM-17* A disintegrin and metalloproteinase domain-17, *TACE* Tumor necrosis factor-alpha converting enzyme, *TNF-α* Tumor necrosis factor alpha, *TGF-α* Transforming growth factor alpha

For this experiment, the upstream activators of ERK1/2 were assessed. Consequently, the functional involvement of COX-2 and ADAM-17 (TACE) (a member of the zinc metalloproteinase superfamily) has been investigated in the activation of the ERK1/2 pathway in TST-induced BPH. It is well known that COX-2-derived PGE2 induces ADAM-17 (TACE) activity (Yang and Chang 2018). To achieve this, the ADAM-17 (TACE) sandwich ELISA kit was utilized, which is the most sensitive tool for detection of ADAM-17 (TACE) activity (Trad et al. 2011; Yousef et al. 2020). It can be seen from **Fig.** 4a that the **TST-treated rats** have approximately **1.7 times** more ADAM-17 (TACE) activity than control rats. As an additional proof, we identified the potential effect of high metalloproteinase activity of ADAM-17 (TACE) on its substrates (*e.g.,* TNF-α and TGF-α). As shown in **Fig.** 4**b** and **c,** **TST-treated rats** had a marked increase in both TNF-α (by **66%**) and TGF-α (by **1.2 times**) compared with **control rats**.

Surprisingly, the pre-treatment with 20 mg/kg/day celecoxib (**CXB-20 plus TST**) significantly decreased ADAM-17 (TACE) activity (by **50%**) compared with **TST-treated rats**. It is interesting that ADAM-17 (TACE) activity was restored to its normal ratio in the **CXB-20 plus TST** group; however, the improvement was smaller in the **CXB-10 plus TST** group. Moreover, the modulated metalloproteinase activity of ADAM-17 (TACE) is reflected on its substrates (*e.g.,* TNF-α and TGF-α). As illustrated in **Fig.** 4**b** and **c**, rats in the **CXB-20 plus TST** group have significantly lower TNF-α (by **34%**) and TGF-α (by **47%**) activity compared with **TST-treated rats**.

### COX-2-induced ERK1/2 phosphorylation is mediated via EGFR in testosterone (TST)-induced BPH

**Fig. 5:**
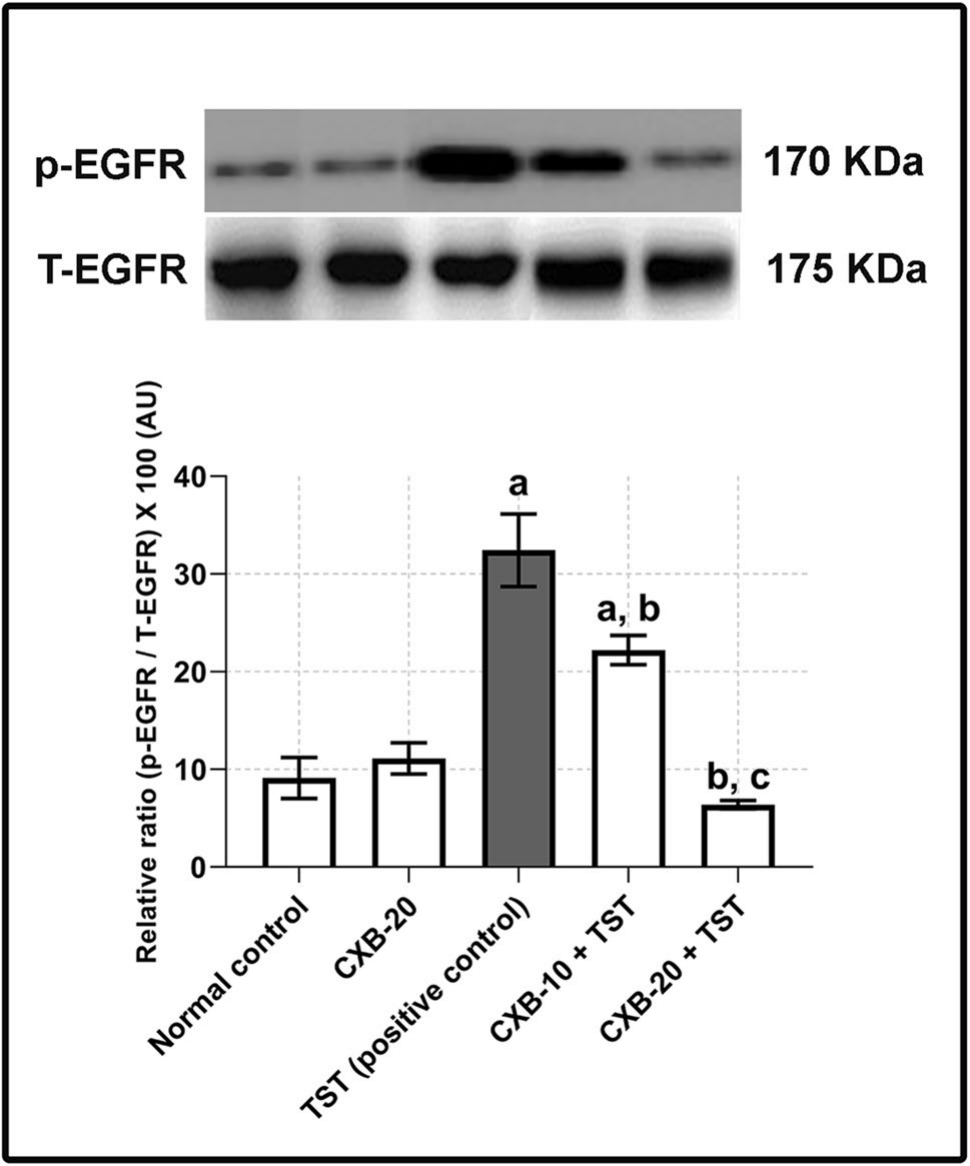
**TST-induced COX-2 activates ERK1/2 pathway via phosphorylation of EGFR in BPH. Assessment of phosphorylation of EGFR using western blotting technique:** Prostate content of p-EGFR/T-EGFR is significantly improved with co-administration of CXB. It is apparent that inhibition of COX-2 leads to reduce phosphorylation of EGFR. The data are provided as mean ± SEM (*n* = 6). ^a^ significant versus *Control*; ^b^ significant versus *TST*; ^c^ significant versus *CXB-10* + *TST* at *P* < 0.05. *CXB* Celecoxib, *TST* Testosterone, *EGFR* Epidermal growth factor receptor

Transforming growth factor alpha (TGF-α) has long been recognized as one of the most effective EGFR ligands (Duffy et al. 2009; Schumacher and Rose-John 2021). There was a clear trend in of increasing TGF-α activity in TST-induced BPH. Therefore, we analyzed the potential impact of high TGF-α activity on EGFR. The western blotting analysis, presented in **Fig.** 5, shows that the phosphorylation of EGFR was strongly increased in **TST-treated rats** above **control rats**. Furthermore, the quantitative analysis in the lower panel of **Fig.** 5 shows a **2.5-fold** increase in EGFR phosphorylation in **TST-induced BPH**.

Following the concomitant use of 20 mg/kg/day celecoxib (**CXB-20 plus TST**), a significant drop was observed in the phosphorylation of EGFR (**Fig.** 5). As shown in the lower panel of **Fig.** 5, rats in the **CXB-20 plus TST** group had decreased EGFR activity (by **80%**) compared with **TST-treated rats**, with a less dramatic change in the **CXB-10 plus TST** group.

### COX-2-induced prostatic hyperplasia is functionally correlated with the intracellular imbalance between proliferative and apoptotic markers in testosterone (TST)-induced rats

**Fig. 6:**
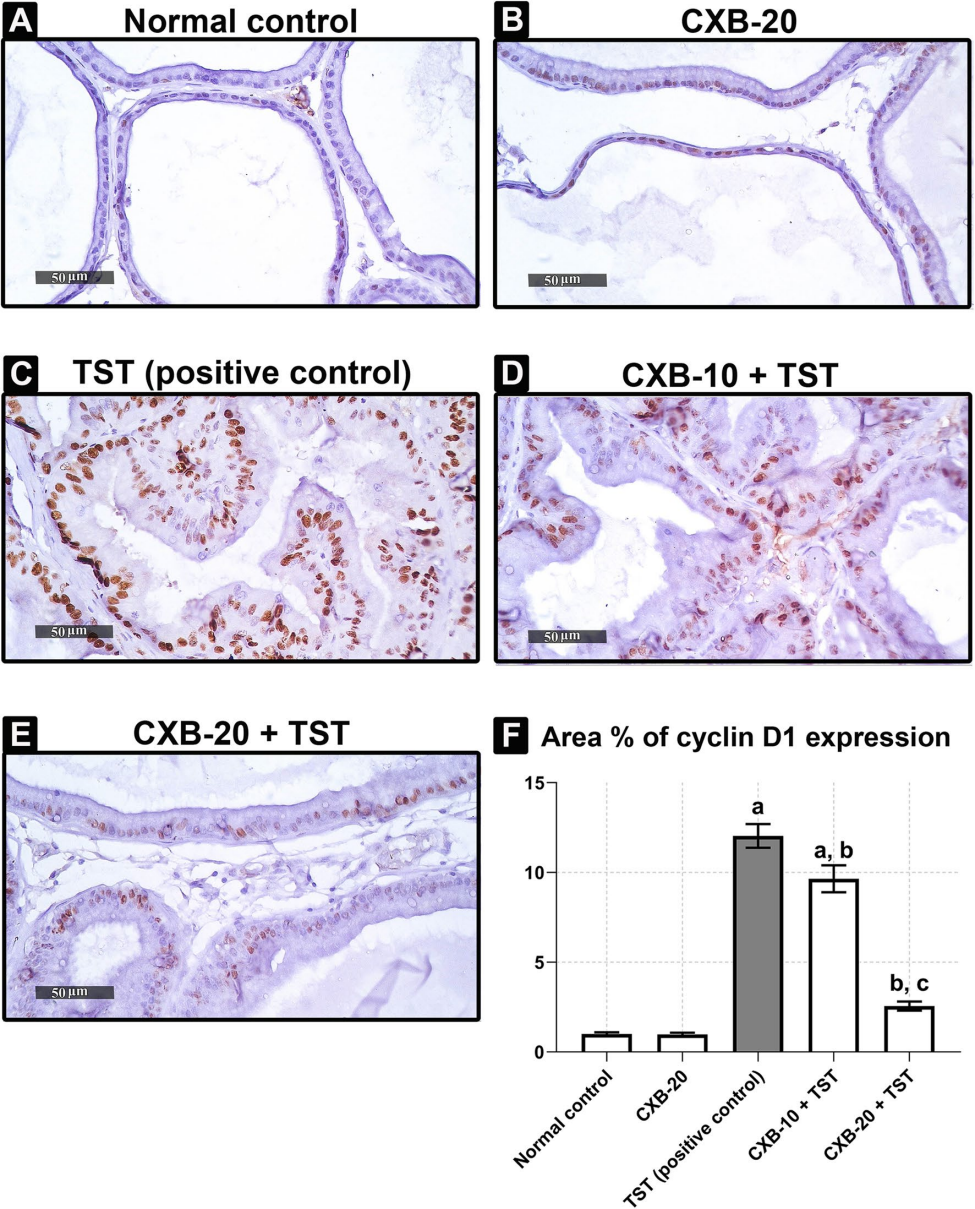
**TST-induced cyclin D1 is abrogated with inhibition of COX-2. Immunohistochemical analysis of rat’s prostate (Scale bar = 50 μm):** **a** **Control** sections demonstrate normal expression of cyclin D1. **b** **Treatment with 10 mg of CXB alone** showing almost the same normal expression of cyclin D1. **c** **Induction of BPH with TST** showing marked high expression of cyclin D1 (*brown color*). **d** **Treatment with 10 mg of CXB** showing almost the same expression of cyclin D1 (*brown color*) as **TST-induced group**. **e** **Treatment with 20 mg of CXB** showing significant improvement of cyclin D1 expression. **f** **Quantitative analysis of cyclin D1 expression** showing normalization of cyclin D1 expression with **CXB-20–treated**. The data are provided as mean ± SEM (*n* = 6). ^a^significant versus *Control*; ^b^significant versus *TST*; ^c^significant versus *CXB-10* + *TST* at *P* < 0.05. *CXB* Celecoxib, *TST* Testosterone

The question in the current experiment was to determine if there was an imbalance between proliferative and apoptotic signals in TST-induced BPH. As shown in **Fig.** 6, Cyclin D1 was highly expressed (in *brown color*) in **TST-treated rats** and quantified as approximately **11 times** (**Fig.** 6**f**) higher than in **control rats**. In contrast, COX-2 inhibition restores the normal balance between proliferative and apoptotic signals. As illustrated in **Fig.** 6**f**, there was no increase in cyclin D1 expression in the **CXB-20 plus TST** group (**79%**) compared with **TST-treated rats**.

**Fig. 7:**
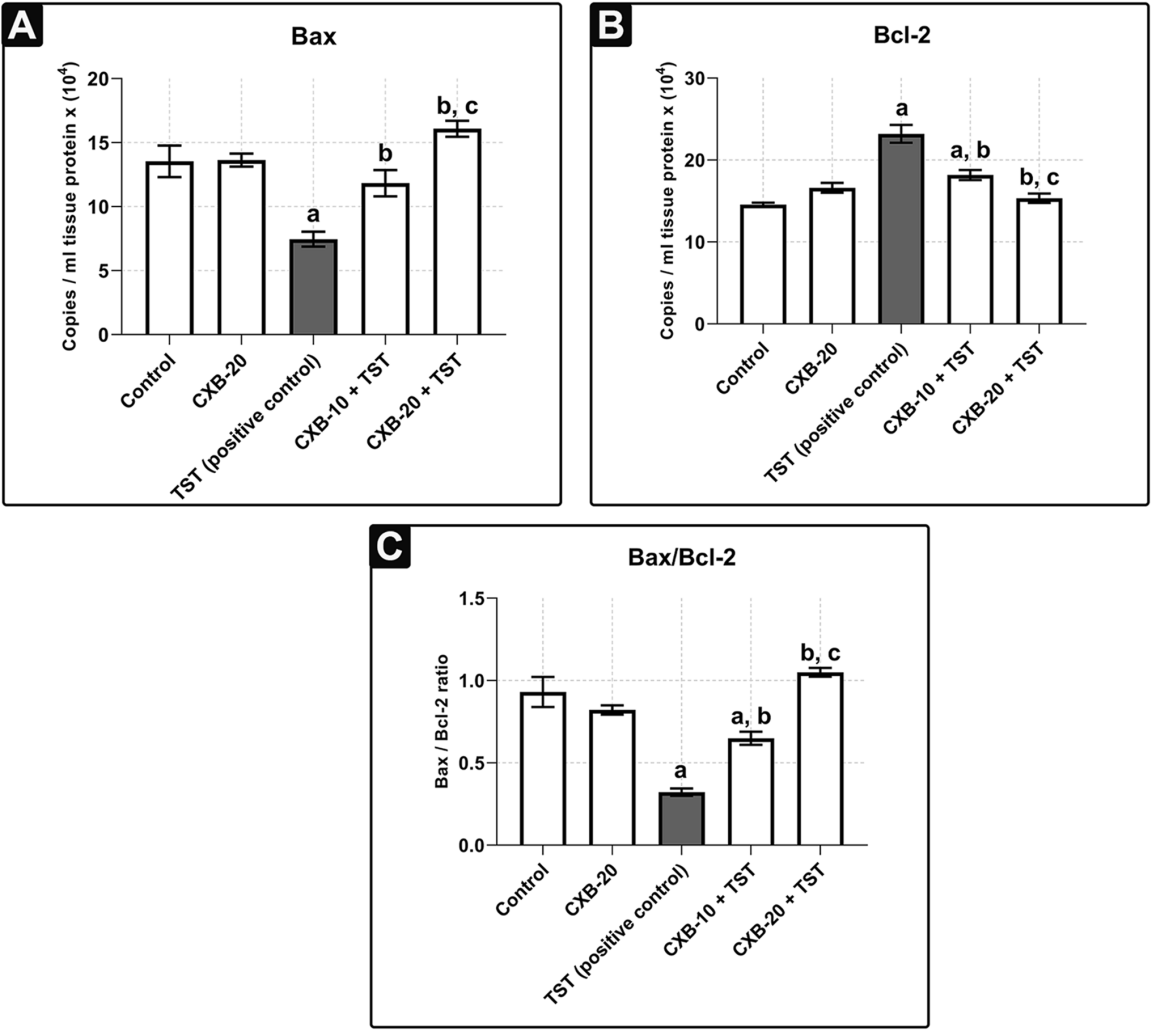
**TST-induced COX-2 disrupts the normal hemodynamic balance between apoptosis (pro-apoptotic Bax) and cell proliferation (anti-apoptotic Bcl-2). Assessment of proliferative versus apoptotic markers using quantitative PCR technique:** Nuclear prostatic content of pro-apoptotic **Bax** **a**, and anti-apoptotic **Bcl-2** **b** are significantly modulated after administration of CXB. All values are assigned as mRNA copies/ml (× 10^4^). Together, pro-apoptotic **Bax**/anti-apoptotic **Bcl-2** ratio **c** is significantly restored to its normal balance with **CXB-20 treated rats**. The data are provided as mean ± SEM (*n* = 6). ^a^significant versus *Control*; ^b^significant versus *TST*; ^c^significant versus *CXB-10* + *TST* at *P* < 0.05. *CXB* Celecoxib, *TST* Testosterone *Bax* Bcl-2 Associated X, *Bcl-2* B-cell lymphoma 2

Further analysis was determined by qRT-PCR (**Fig.** 7**a** and **Fig.** 7**b**), which revealed the high mRNA expression of anti-apoptotic Bcl-2 (**59%** more than the **control**) and significantly lower mRNA expression of pro-apoptotic Bax (**45%** less than **control**) in **TST-treated rats**. Furthermore, mRNA expression of Bax was elevated in the **CXB-20 plus TST** (**116%**) compared with **TST-treated rats** (**Fig.** 7**a**). In addition, the mRNA expression of anti-apoptotic Bcl-2 was reduced (**34%**) in the **CXB-20 plus TST** compared with **TST-treated rats** (**Fig.** 7**b**).

Collectively, the general trend in **TST-induced BPH** is for a lower ratio of pro-apoptotic Bax per anti-apoptotic Bcl-2 (**65%**) compared with the **control** group (**Fig.** 7**c**). On the other hand, COX-2 inhibition by 20 mg/kg/day celecoxib (**CXB-20 plus TST**), reversed the imbalance between proliferative and apoptotic signals by inducing a higher ratio of pro-apoptotic Bax per anti-apoptotic Bcl-2 compared with **TST-induced BPH** (**Fig.** 7**c**). The fewest changes were detected in the **CXB-10 plus TST** group.

## Discussion

The BPH is the fourth most prevalent male diagnosis (Pizzorno et al. 2016). In general, the prevalence of BPH increases with age. There is an overall prevalence of 2%–25% with a further increase to 45% in older patients. From every 1000 persons, it is estimated that 9–41 patients are affected per year, with high treatment expenditure (USD 3–10 billion/year). Several factors, such as infectious agents, urine retrograde, metabolic syndrome, aging, and autoimmune disorders, may be involved in the initiation and progression of BPH nodules. All of these triggers participate in the activation of inflammatory signaling pathways, which ultimately results in prostatic enlargement (De Nunzio et al. 2020). In agreement with this concept, the current study evaluated the association between inflammation and hyperplasia in BPH. To this end, we selected the induction of BPH using testosterone (TST) in male rats, which is the most accepted experimental model of BPH in elderly patients (Altavilla et al. 2012; Abo-Youssef et al. 2020; Eid and Abdel-Naim 2020; Almukadi et al. 2021; Zhao et al. 2021).

Generally, TST is converted to dihydrotestosterone (DHT) via the 5-alpha-reductase enzyme. DHT is five times more active than TST. Consequently, DHT is able to induce prostatic hypertrophy by stimulating production of various growth factors (Madersbacher et al. 2019). As an additional pathway, TST is further bio-transformed into estradiol (the potent form of estrogen) through the action of CYP19/aromatase (Santen et al. 2009). Interestingly, the negative impact of estradiol on abnormal prostate enlargement was previously determined (Prins et al. 2006). As mentioned previously, BPH is well characterized with the presence of high levels of inflammatory mediators concurrently with prostatic hyperplasia (Abo-Youssef et al. 2020; Afify et al. 2020). However, the molecular mechanism through which prostatic inflammation induces hyperplasia has remained uncertain.

To prove this hypothesis, celecoxib (CXB) was employed prophylactically in the current study for three reasons. First, it is a standard COX-2 inhibitor (i.e., an inhibitor of inflammation) (Goldenberg 1999; Dougados et al. 2001). Second, the effectiveness of CXB (200 mg) as an anti-inflammatory in human BPH patients has also been shown in clinical trials (Falahatkar et al. 2008; Goodarzi et al. 2011). Third, CXB is proposed to also block the synthesis of IL-6 and prostaglandins, in particular prostaglandin E2 (PGE2), which is the main mediator in BPH (Anderson et al. 1996; Hinson et al. 1996). Consequently, CXB is the best choice for the validation of the current hypothesis. Based on the dose translation studies of *Nair *et al*.*, the current preclinical study utilized 10 and 20 mg/Kg of CXB to best cover the human effective range experienced by *Goodarzi *et al. (Goodarzi et al. 2011; Nair et al. 2018). It is interesting to mention that COX-2 inhibitory action begins to weaken with daily doses above 50 mg/kg and entirely abates above 100 mg/kg (Niederberger et al. 2001). On safety, CXB has an FDA-boxed warning regarding possible adverse cardiovascular (C.V.S.) effects, notably heart attacks and strokes (Nissen et al. 2016; Barcella et al. 2019). In fact, there are some controversies about this issue. For instance, *Barcella *et al*.*, discussed that even low doses of CXB have been associated with a significant C.V.S. risk (Barcella et al. 2019). However, *Nissen *et al*.*, shown through clinical research that the substantial C.V.S risk only applies to doses more than 200 mg (Nissen et al. 2016). Interestingly, a similar dose of CXB was thoroughly examined and found to be safer for the entire G.I. tract than standard NSAIDs (Chan et al. 2010; García-Rayado et al. 2018). It is noteworthy that a benefit/risk ratio should be considered for prescribing CXB in patients at high C.V.S. risk (García-Rayado et al. 2018). Consistently, the present study was applied on rat equivalent dose (20 mg/Kg) to maximize the therapeutic efficacy with the least possible side effects.

Indeed, although TST normally declines with advancing age, intra-prostatic levels of DHT are maintained at normal levels. Elderly patients have a high incidence of coexisting clinical disorders that may further contribute to elevated levels of cytokines and immune responses in a “multi-hit” scenario. Accordingly, all of these factors may deviate the action of TST/DHT toward marked elevation of growth factors, including keratinocyte growth factor, EGF, insulin-like growth factors, and TGF-α. Ultimately, this pathway prompts the induction of stromal cell proliferation (Vignozzi et al. 2014). In addition, the same factors may be implicated in prostatic enlargement following abuse or long-administration of anabolic steroids in young athletes (Kanayama et al. 2008, 2010). Moreover, estrogen levels concurrently increase or remain constant. This provides strong support for the second scenario, which revealed the occurrence of BPH as a result of hormonal imbalance between androgens and estrogens in elderly patients. Regardless of TST level, BPH as a metabolic disorder may be essentially induced via TST, but it not crucial (Vignozzi et al. 2014).

The current study used a sequential process. Initially, the possible involvement of the NF-κB inflammatory pathway was explored in BPH. Indeed, NF-κB is a crucial component in a number of immunological and inflammatory processes. NF-κB is an inducible complex protein consisting of the p50 (NF-κB1) and p65 (RelA) subunits. When NF-κB is present in the cytoplasm, it is inactivated via sequestration with IκBα (the inhibitory protein). *Gonzales *et al*.* previously demonstrated that DHT binds to the intracellular androgen receptor (AR), resulting in the formation of a DHT–AR complex. It was also found that the DHT–AR complex increases nuclear translocation of NF-κB through the suppression of IκBα in cerebral blood vessels (Gonzales et al. 2009). Then, NF-κB binds to sequence-specific DNA binding sites and subsequently induces transcription of the gene encoding COX-2 (Liu et al. 2017). The results of the current study in BPH agree with these observations. Furthermore, several authors confirmed the inhibition of DHT-mediated NF-κB in the presence of anti-androgen therapy (Gonzales et al. 2009; Hsu et al. 2021). As CXB is a potent inhibitor of COX-2 (Goldenberg 1999) and NF-κB (Funakoshi-Tago et al. 2008), the current use of 10 and 20 mg/kg/day CXB effectively reduces the levels of COX-2 consistent with inhibition of NF-κB. Inflammation in BPH is therefore characterized primarily by COX-2. This observation is consistent with previous notation (Chughtai et al. 2011). It can therefore be assumed that PGE2 will also be induced.

For confirmatory purposes, the histopathological examination revealed severe deterioration of cellular architecture characterized by hallmarks of congestion, edema, and inflammation in BPH. The accumulated evidence agrees these results (Abo-Youssef et al. 2020; Afify et al. 2020). One possible explanation for this might be that inflammatory responses involve the translocation of phospholipase A_2_ from the cytosol to the nuclear membrane where the enzymatic hydrolysis of phospholipids takes place (Leslie 2004). Simultaneously, inflammation also induces transcription of the COX-2 and microsomal prostaglandin synthase E-1 (mPGE-1) genes. COX-2 acts on arachidonic acids (byproducts of phospholipids), producing prostaglandin H2, which is further converted to PGE2 (Morita 2002). The most clinically relevant findings are the strong inhibitory actions of CXB, which were clearly explored in histopathological examination. As further confirmation, the levels of PGE2 were markedly induced in the present study, concurrently with the high levels of COX-2. In contrast, the levels of both COX-2 and PGE2 are significantly suppressed in the presence of CXB. These results further support the idea that PGE2 is a principal mediator in inflammatory disorders (Park et al. 2006). Consequently, it was proposed that COX-2-derived PGE2 upregulates a positive feedback mechanism to induce expression of IL-6 in adjuvant arthritis (Anderson et al. 1996; Hinson et al. 1996). The current results mirror those of the previous studies that have examined the relationship between COX-2 and IL-6.

The next question in the current study was whether COX-2-derived PGE2 is essentially to the pathogenesis of BPH. The current findings corroborate the ideas of *Pai *et al*.*, who suggested that PGE2 can induce gastric and intestinal hypertrophy. In addition, these results further support the involvement of PGE2 in colon cancer (Pai et al. 2002; Shao et al. 2003). Interestingly, the current results strengthen this idea by COX-2 inhibition. In the **CXB-10** and **CXB-20** groups, prostatic enlargement was markedly improved, and the **CXB-20** group showed the best improvement.

The current results confirm the link between COX-2 and prostatic hypertrophy. Based on this, this study performed a thorough investigation of the mechanistic relevance of prostatic hypertrophy. Western blotting clearly showed the increase in ERK1/2 phosphorylation with COX-2 overexpression. The involvement of the ERK1/2 pathway in BPH is perhaps the most clinically significant finding in this study. Another finding was the marked inhibition of ERK1/2 phosphorylation in **CXB-treated animals**. In agreement with *Ansari* et al*.*, COX-2-derived PGE2 induces cell proliferation, which may progress to tumor in keratinocytes (Ansari et al. 2008). To further prove the implication of ERK1/2 pathway in BPH, this study investigated upstream and downstream signals involved in this pathway.

There are several mechanisms for the induction of trophic action via COX-2-induced PGE2 (Yang and Chang 2018). One of them is via activation of metalloproteinase activity, which was the main focus of the current study. Several studies have demonstrated the role of ADAM-17 (TACE) as a cleaving enzyme in the activation of TNF-α and TGF-α (high-affinity EGFR ligands) (Duffy et al. 2009; Schumacher and Rose-John 2021). PGE2 overexpression causes ecto-domain shedding of TNF-α and TGF-α (the highest affinity EGFR ligand) via ADAM-17 (TACE). Active TGF-α binds to EGFR, causing it to dimerize and phosphorylate, activating the EGFR–ERK1/2 signaling cascade (Pai et al. 2002; Wee and Wang 2017). ERK1/2 activation plays an important role in inducing proliferative (*e.g.,* cyclin D1 and anti-apoptotic Bcl-2) signals and inhibiting apoptotic signals (Bax) (Bonnefoy-Berard et al. 2004). Based on the findings of *Abo-El Fetoh *et al*.*, phosphorylated ERK (p-ERK1/2) trans-locates from the cytosol to the nucleus (Abo-El Fetoh et al. 2020). p-ERK1/2 induces the expression of cell proliferation genes (i.e., cyclin D1 and anti-apoptotic Bcl-2) and abrogates the expression of pro-apoptotic Bax. Finally, the normal cell cycle is disrupted (Bonnefoy-Berard et al. 2004; Fu et al. 2019). At mRNA level, pro-apoptotic Bax, anti-apoptotic Bcl-2, and the Bax/Bcl-2 ratio were dysregulated compared with **control samples**. These results mirror those of the previous studies that have examined the dysregulation in pro-apoptotic Bax and anti-apoptotic Bcl-2 (Abdel-Naim et al. 2018; Abo-Youssef et al. 2020). Surprisingly, no differences were found when the protein levels of pro-apoptotic Bax and anti-apoptotic Bcl-2 were detected (Almukadi et al. 2021; D’Amico et al. 2021; Hsu et al. 2021). This implies that the normal hemodynamic balance of the prostate may be disrupted and further directed toward hyperplasia.

The activation of cAMP/protein kinase A pathway is considered another mechanism for PGE2-induced hypertrophy, and leads to an increase in the expression of amphiregulin (another EGFR ligand) (Shao et al. 2003). The activation of the intracellular Src-mediated pathway, the third mechanism, is independent of the release of extracellular EGFR ligand (Buchanan et al. 2003). Together, it is believed that all of these mechanisms activate the positive feedback loop to further increase gene expression of COX-2 and PGE2, leading to greater induction of the EGFR–ERK1/2 pathway (Dannenberg et al. 2005).

## Conclusion

**Fig. 8:**
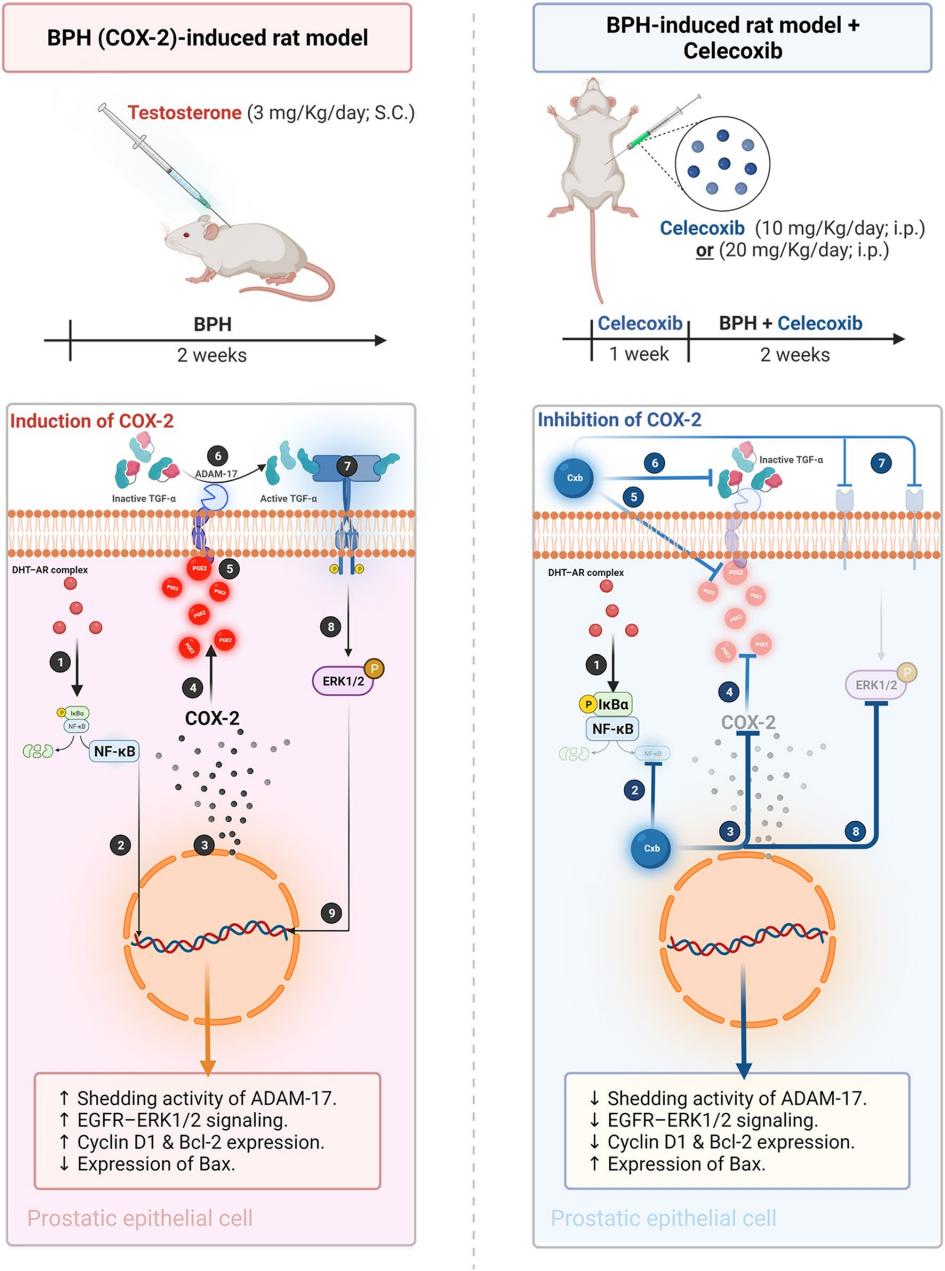
**Graphical diagram of functional correlation between inflammation and hyperplasia in TST-induced BPH. Cycloxygenase-2 (COX-2) upregulates EGFR–ERK1/2 signaling cascade via stimulation of PGE2-induced ADAM-17 and consequent shedding of TGF-α:** The left panel (in *red color*) shows that **TST-induced BPH** stimulates the nuclear translocation of NF-κB [**1–2**] resulting in increasing gene transcription of COX-2 [**3**] and subsequent PGE2 [**4**]. PGE2 trans-activates EGFR via intracellular activation of ADAM-17 (TACE) [**5**] leading to shedding of TGF-α [**6**]. Consequently, active TGF-α phosphorylates EGFR and induce ERK1/2 phosphorylation [**7–8**]. Together, the nuclear translocation of p-ERK1/2 [**9**] induced prostatic hyperplasia via enhancing gene expression of cyclin D1 and anti-apoptotic Bcl-2 in consistent with downregulation of pro-apoptotic Bax expression. In the contrast, the panel in the right corner (in *blue color*) shows the potential effect of COX-2 inhibition in TST-induced BPH

The present study uncovers the functional correlation between COX-2–PGE2 and ADAM-17 in BPH, which may be a future target for the management of BPH while preserving the current level of testosterone and mitigating interference from anti-androgen therapy. In summary, COX-2 induces the ERK1/2 pathway via PGE2–ADAM-17-catalyzed shedding of TGF-α in testosterone-induced BPH (**Fig.** 8). Furthermore, the current study suggests a novel treatment option (i.e., CXB) that is suitable not only for nocturia induced by BPH but also for prostatic hyperplasia.

## Data Availability

The data that support the findings of this study are included within the manuscript or the supplementary data.

## Abbreviations

COX-2: Cyclooxygenase enzyme isoform 2
PGE2: Prostaglandin E2
NF-κB: Nuclear factor kappa-light-chain-enhancer of activated B cells
IL-6: Interleukin 6
ADAM-17/TACE: A disintegrin and metalloproteinase domain-17/Tumor necrosis factor-alpha converting enzyme
TGF-α: Transforming growth factor alpha
TNF-α: Tumor necrosis factor alpha
EGFR: Epidermal growth factor receptor
ERK1/2: Extracellular regulated kinase protein 1 and 2
Bcl-2: β-Cell lymphoma 2
Bax: Bcl-2-associated X protein
